# A High-Efficiency Multispectral Filter Based on Plasmonic Hybridization between Two Cascaded Ultrathin Nanogratings

**DOI:** 10.3390/molecules24112038

**Published:** 2019-05-28

**Authors:** Bo Zhao, Zhenfen Huang, Jianjun Yang, Lei Zhang, Rajagopal S. Joshya, Chunlei Guo

**Affiliations:** 1 Department of Electronic Information and Physics, Changzhi University, Changzhi 046011, Shanxi, China; zy828522@163.com (B.Z.); siyu2009-h@163.com (Z.H.); zhanglei880221@163.com (L.Z.); 2 The Guo China-US Photonics Laboratory, State Key Laboratory of Applied Optics, Changchun Institute of Optics, Fine Mechanics and Physics, Chinese Academy of Sciences, Changchun 130033, Jilin, China; Joshyashyamala@gmail.com; 3 The Institute of Optics, University of Rochester, Rochester, New York, NY 14627, USA

**Keywords:** surface plasmon polariton (SPP), metal-insulator-metal (MIM) structures, leaky and bound mode, waveguide mode, multispectral filter, plasmonic hybridization

## Abstract

Overcoming the disadvantages of low transmission and broad peak bandwidth of previously reported plasmonic color filters, a high-efficiency multispectral plasmonic color filter is theoretically proposed with two cascaded ultrathin metallic nanogratings separated by two heterogeneous dielectric layers, and its optical properties are theoretically investigated using the finite-difference time-domain method. The transmission spectrum presents three near-unity peak bands accompanied with three near-null dip bands adjacent around them. Both transmission efficiencies of above 90% and ultranarrow peak bandwidth of 20 nm are achieved in the visible regime. The peak band positions can be flexibly tailored by varying the structural parameters. The filter selects the visible color with high signal noise ratio at the peak bands. The outstanding spectral properties of this filter indicate significant improvement for the high-accuracy color filtering and multispectral imaging applications. The simulated near-field electromagnetic distributions suggest that the excitation of the hybrid antisymmetric surface plasmon polariton (SPP) leaky mode and metal-insulator-metal waveguide modes are responsible for the peak transmission bands, while the formation of the hybrid SPP bound modes confined on the bottom nanograting makes the dip transmission bands, all of which are the consequence of the plasmonic hybridization between the two neighboring metallic nanogratings.

## 1. Introduction

Since the pioneering report of the extraordinary transmission (EOT) phenomenon by Ebbesen [1], metallic subwavelength structures have been an attractive subject of intense study due to the capabilities for manipulating the light at subwavelength scales by the excitation of surface plasmon polariton (SPP) [2,3,4,5,6], which is essentially a hybrid electromagnetic mode originating from the coupling of the light with the collective oscillations of free electrons at the metal/dielectric interface. The highly-tunable enhanced transmission peaks for a single-layer optically opaque metallic film perforated with subwavelength holes/slit arrays are capable of selective filtering of the light in the optical regime, which have potential applications in spectral imaging, optical sensing, color printing, digital displays, and so on [7,8,9,10,11,12,13]. However, the broad peak bandwidth and the low transmission features of such single-layer nanostructured films severely restrict their commercial and practical applications. Moreover, the large longitudinal volume size does not fulfill the requirement of the ultracompact integrated optical systems. Some attempts to design the ultrathin nanostructured metal films as subtractive color filters based on the suppressed transmission have experimentally improved the absolute transmission efficiency over 60%, but the visible spectrum cannot be accurately distinguished due to the relative broad bandwidth [14]. Recently, obtaining a narrow peak bandwidth and high transmission efficiency have also been experimentally reported for plasmonic color filters, which consist of the multilayer stacks with metallic nanostructured films and dielectric layers to support a multitude of hybridized SPP modes [15,16,17,18]. For instance, based on a subwavelength periodic metal-insulator-metal (MIM) stack, a nanoslit array on the transparent film of magnesium fluoride (MgF_2_) was utilized as plasmonic color filtering to achieve peak transmission efficiency over 60%, with pass bandwidth larger than 100 nm in the visible regime [15]. A plasmonic color filter constructed by the free-standing MIM stack slit array in the Au/Si_3_N_4_/Au film had significantly improved the transmission efficiency up to 90% in near-infrared regime but with broad peak bandwidth of approximately 200 nm [16]. A hexagonal array of subwavelength holes in a thin CMOS-compatible MIM stack was designed to selectively filter the light in the visible regime, with the measured peak transmission efficiency up to 60% and narrow bandwidth of 45~55 nm [17]. Through five alternative layers of Ag and SiO_2_ as a multilayer slot-mode plasmonic filter (MSPF) in the visible regime, it is capable of achieving a single peak bandwidth as narrow as 17 nm and 40% transmission efficiency [18]. Nonetheless, all these MIM structures still have the problems of either relative low transmission efficiency or relative broad bandwidth. Multiple spectral plasmonic color filters that were theoretically proposed and composed of two plasmonic nanoparticle arrays (such as nanosphere, nanocore–shell, nanocylinder, nanoellipsoid, etc.) on the surfaces of a seamless ultrathin metal film or inserted between two seamless ultrathin metal films achieved the highest peak transmission efficiency of 86% and narrowest bandwidth of 25 nm [19,20,21,22,23]. However, complex multilayer designs composed of two-dimensional nanoparticle arrays are required.

In this paper, we theoretically propose and demonstrate a multispectral plasmonic color filter with near-perfect peak transmission efficiency and ultranarrow bandwidth by utilizing the two-dimensional finite-difference time-domain (FDTD) method. The designed structure includes two ultrathin metallic nanogratings cascaded with two heterogeneous dielectric layers, an air/vacuum layer, and a silicon dioxide dielectric layer, which results in three near-unity enhanced peak transmission bands and three near-null suppressed dip transmission bands in the visible and near-infrared regime. Especially, the peak bandwidths in the visible regime can be as narrow as 20 nm. Deep insights into the physical mechanisms of the observations are obtained from the calculations of the electromagnetic (EM) field distributions. By varying the geometric parameters, the proposed multispectral plasmonic color filter can accurately separate the visible light into many color channels based on the ultranarrow peak transmission bandwidth, which allow its spectral resolution imaging and sensing analysis to further surpass the human eye’s capability, and pave a noninvasive way for material identification, target location, quality monitoring, safety diagnosis, astronomy and space surveillance, and particularly for the acquisition of optical high-resolution spectral information [24,25,26].

## 2. Methods

**Figure 1:**
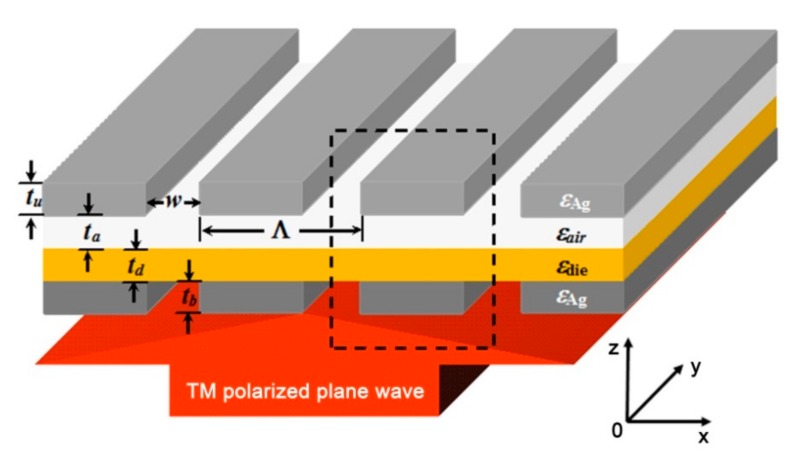
A schematic illustration of the proposed multispectral plasmonic color filter, which is composed of two aligned metallic ultrathin nanogratings cascaded with two heterogeneous layers, an air layer (separation) of thickness *t*_a_ and a SiO_2_ dielectric layer of thickness *t*_d_. Two metallic nanogratings have the identical period of Λ and slit width of *w*, *t*_u_ and *t*_b_ donate the thicknesses of the upper and bottom nanogratings, respectively.

The configuration of the proposed plasmonic multilayer structure is schematically illustrated in Figure 1, which is designed to act as a multispectral color filter in the visible regime. Two silver ultrathin nanogratings are longitudinally cascaded with an air layer of thickness *t*_a_ and a silicon dioxide (SiO_2_) layer of thickness *t*_d_, wherein the bottom nanograting is adhered under the SiO_2_ dielectric layer and the upper one is suspended atop the air layer (separation), resulting in asymmetric dielectric environments for both sides. The whole structure is free-standing and embedded in air. The two nanogratings have the same period of Λ and slit width of *w*, but with different thicknesses of *t*_b_ and *t*_u_, respectively. The gap between two cascaded nanogratings can be considered as a series of horizontal MIM waveguides. The dielectric permittivity of SiO_2_ is *ε_die_* = 2.25. The relative permittivity of silver is described by the Drude model: $\varepsilon_{Drude}=\varepsilon_{\infty }-\omega_{p}^{2}/\omega \left(\omega -i\gamma \right)$ where the constants of *ε*_∞_ = 3.7, *ω*_p_ = 1.3673 × 10^16^ rad/s and *γ* = 2.7325 × 10^13^ rad/s are extracted from the experimental data [27]. The FDTD method was employed to calculate the transmission properties of the multispectral plasmonic filter color. The calculated region is fixed as a period cell and marked by a black dotted square box in Figure 1. The top and bottom boundaries of the calculated region are set as perfectly matched layers (PMLs), while the left and right are periodic boundary conditions. The calculated wavelengths are λ = 400~2000 nm, which range from the visible to the near-infrared regime. A monochromatic plane wave is set as the light source for calculating EM field distributions.

Under the irradiation of a TM polarized plane wave (with electric field perpendicular to the nanograting slit), SPP modes can be excited on the upper and bottom nanogratings, respectively [28]. Due to the field overlapping between them, the SPP hybridization effect introduces new coupled modes based on the structural parameters of the MIM waveguide and the incident wavelengths, which are devoted to enhancing or suppressing the light transmission through the filter. To explore the performance of the designed plasmonic color filter, the response of the transmission spectrum to the variation of the structural parameters, including the grating thickness *t*_u_ and *t*_b_, period Λ and the air separation *t*_a_ are investigated. The new hybrid SPP modes formed at different wavelengths are demonstrated by the calculated spatial distribution of the EM fields.

## 3. Results and Discussion

### 3.1. Trasmission Spectrum

**Figure 2:**
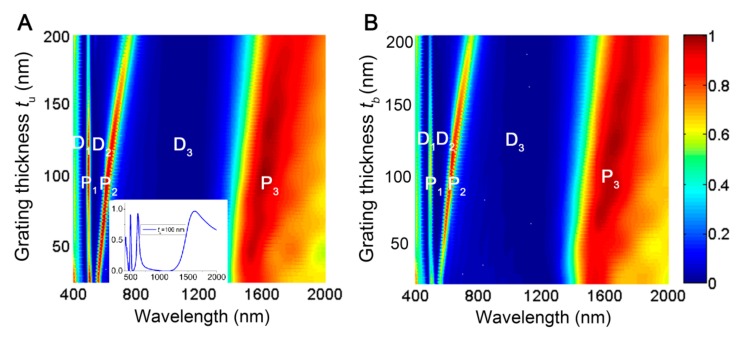
Transmission spectra of the multispectral plasmonic color filter as a function of (**A**) the thickness of the upper nanograting *t*_u_ with a fixed *t*_b_ = 30 nm; and (**B**) the thickness of the bottom nanograting *t*_b_ with a fixed *t*_u_ =100 nm. Other parameters are set as Λ = 500 nm, *w* = 150 nm, *t*_d_ = *t*_a_ = 50 nm. The inset picture in Figure 2A shows the spectrum profile at *t*_u_ = 100 nm and *t*_b_ = 30 nm.

The calculated transmission spectra of the multispectral plasmonic color filter with respect to the wavelengths and the upper nanogratings thicknesses *t*_u_ is shown in Figure 2A, where the fixed bottom nanograting thickness is *t*_b_ = 30 nm, and other geometric parameters are given by Λ = 500 nm, *w* = 150 nm, and *t*_d_ = *t*_a_ = 50 nm. Obviously, the transmission spectra present two ultranarrow peak transmission bands in the visible regime, which are respectively marked by P_1_ and P_2_. On their two lateral sides, there are three near-null dip transmission bands that are denoted by D_1_, D_2_, and D_3_. Notably, the dip bands of D_1_ and D_2_ in the visible regime possess the same full-width at half-maximum (FWHM) at the scale of tens of nanometers as that of the peak bands P_1_ and P_2_, while the dip band D_3_ in the near infrared region has an FWHM of hundreds of nanometers. Besides, there is a nonresonant peak transmission band, P_3_, with a FWHM of hundreds of nanometers located in the red side of D_3_. Here, “nonresonant” means that the wavelength of the peak band P_3_, which is about 3.5 times larger than the size of the periodic cell, does not satisfy the SPP waveguide resonance conditions. As *t*_u_ increases, the wavelength of the peak band P_1_ always remains unchanged at around 495 nm, as shown in Figure 2A, while its transmission efficiency first rises and then rapidly drops, and reaches the maximum value of above 90% with a FWHM as narrow as 20 nm at around *t*_u_ = 100 nm (as shown by the inset picture); for the peak band P_2_, the wavelength position displays a nonlinear redshift with the increase of *t*_u_, and its transmission efficiency varies in a nonmonotonic way and reaches 93% at *t*_u_ = 100 nm with a FWHM of 40 nm; the peak band P_3_ with the broad FWHM linearly shifts toward the larger wavelength with increasing *t*_u_, but its transmission efficiency has no obvious fluctuate and remains above 90%. As *t*_u_ increases, the wavelengths of the dip bands D_2_ and D_3_ display redshift and the FWHM of D_2_ obviously becomes broader; the transmission efficiency of three dip bands D_1_, D_2_, and D_3_, respectively, are suppressed to 0.2%, 0.3%, and 0.2% at *t*_u_ = 100 nm, and remain at nearly zero with the increase of *t*_u_; both the position of the dip band D_1_ and the bandwidths of the dip bands D_1_ and D_3_ have no obvious change. It is supposed that the dip transmission behaviors have adjustments relative to the redshift of the peak bands with the increase of *t*_u_.

In addition, the evolution of the calculated transmission spectra with varying bottom nanograting thicknesses *t*_b_ at *t*_u_ = 100 nm is shown in Figure 2B, being nearly identical to the case of Figure 2A, except for the declined transmission efficiency of the peak bands P_1_ and P_2_. These results indicate that the multispectral plasmonic color filter can perform not only improve the transmission behaviors at the peak bands, but also has nearly zero suppression behaviors at the dip bands, once the upper and bottom grating thicknesses are decreased comparable to its skin depth (optically-thin). Most importantly, the ultranarrow FWHM of peak and dip bands in the visible regime allow the accurate filtering of the light’s color, which is very important for the development of practical devices based on multi- and hyper-spectral imaging and sensing.

### 3.2. Spatial Distribution of EM Fields

#### 3.2.1. At Peak Transmission Bands

**Figure 3:**
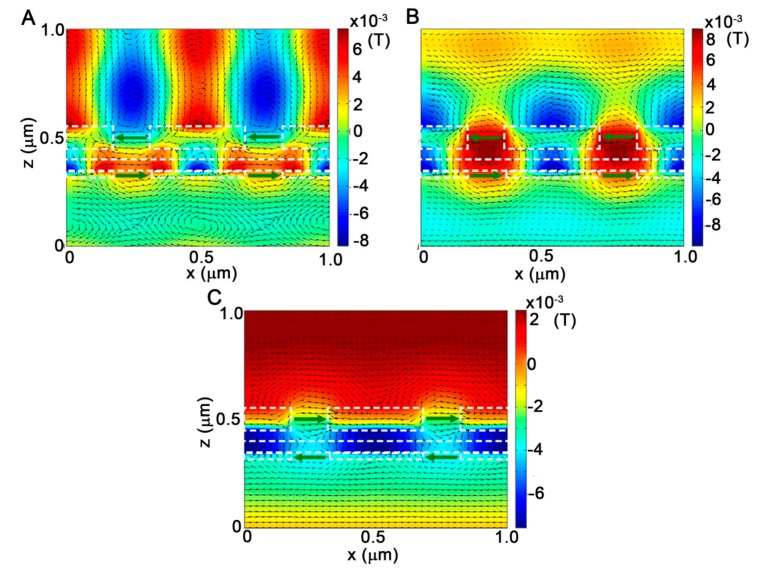
Calculated magnetic field H_y_ (color map), electric displacement (arrow map) distributions for the multispectral plasmonic color filter at the peak transmission bands of (**A**) λ = 495 nm, (**B**) λ = 610 nm and (**C**) λ = 1745 nm. The structural parameters are *t*_u_ = 100 nm, *t*_b_ = 30 nm, *w* = 150 nm, *t*_d_ = *t*_a_ = 50 nm and Λ = 500 nm.

In order to explore the physical origin of the peak transmission bands presented in Figure 2, the corresponding spatial distribution of the EM fields at the situations of *t*_u_ = 100 nm and *t*_b_ = 30 nm are displayed in Figure 3, where the white dashed lines represent the boundaries of different materials to separate the upper metallic nanograting, the air separation, the SiO_2_ layer, and the bottom metallic nanograting. Figure 3A shows the plots of the magnetic field H_y_ (color map) and electric displacement (arrow map) distributions within two periodic cells of the multispectral plasmonic color filter at the peak band P_1_ of λ = 495 nm. It is clearly observed that magnetic fields are localized not only within the MIM waveguide layer, but also on the incident and transmission interfaces of the filter, with 2nd order standing-wave patterns along the x-direction, which are characterized by two field antinodes with the opposite phase formed in one periodic cell. However, there is no obvious SPP cavity mode formed in the vertical slit. Note that in the vertical direction, H_y_ fields within the MIM waveguide layer are in the opposite phase to that on the transmission and incident interfaces, which are subjected to double phase shifting of π vertically across the incident interface to the transmission side. It means that a new hybrid antisymmetric SPP leaky mode is formed due to the hybridization between SPP waves excited on the upper and bottom nanogratings, which is responsible for the peak transmission band. The electric displacements denoted by the black arrows demonstrate that two electric dipoles are respectively induced on the input and output slit portions of the multispectral plasmonic color filter, which play dominant roles in the peak band transmission [16,17]. Upon the excitation of the hybrid antisymmetric SPP leaky mode, the incident plane wave can be optimally coupled into the SPP mode through the bottom nanograting into the MIM waveguide layer, then decouple SPP waves into the plane wave again by the upper nanograting, leading to the peak transmission band P_1_. Since this SPP leaky mode associated with the peak band P_1_ matches the resonant condition on the transmission interface along the x-direction, its corresponding wavelength can be evaluated by:

*λ*_0_ = *n*_eff_ * *λ*_sp_ = *n*_eff_ * Λ, (1)

where $n_{eff}=\sqrt{\varepsilon_{m}\varepsilon_{d}/(\varepsilon_{m}+\varepsilon_{d})}$ is the effective index of SPP wave on the transmission at the interface. It can easily be inferred that the wavelength of the peak transmission associated with the hybrid antisymmetric SPP leaky mode is mainly determined by the periodicity of the multispectral plasmonic color filter, which will be verified by the evolution of the transmission spectra with the structural period in the next section.

The plots of the corresponding EM field distribution at the peak band P_2_ of λ = 610 nm are displayed in Figure 3B. Different from the peak band P_1_, the H_y_ field is mainly concentrated within the horizontal MIM waveguide layer and form a 2nd order waveguide modes along the x-direction within one periodic cell, which is evidenced by the opposite phase of the two modes. In the vertical direction, H_y_ fields in the slits of the upper and bottom nanogratings couple together across the air and dielectric layers to generate a 1st order Fabry–Perot cavity SPP resonance mode, which possesses two mirrors of finite reflection at the end of the slit. In this case, H_y_ fields simultaneously participate in the horizontal waveguide mode and vertical cavity mode. The coupling effect between the MIM waveguide mode and cavity mode makes the peak band P_2_. On the top and bottom metallic nanogratings, two dipoles are respectively generated, as shown by the electric displacements, and dedicated to transferring light through the multispectral plasmonic color filter. The transmission spectrum profile of the multispectral plasmonic color filter is predominantly modulated by the cross-shaped cavities consisting of the horizontal waveguides and the vertical slits. The larger upper and bottom nanograting thickness increases the vertical length of the cross-shaped cavity, resulting in a linear redshift of the peak transmission band P_2_, which accords with the results in Figure 2A,B. On the transmission interface, the H_y_ field is located at the center of the nanograting ridge with a state of resonance. Based on the cross-shaped cavity, the wavelength of the peak transmission band P_2_ can be quantificationally estimated from the expression:

(2) $$\frac{2\pi n_{eff}}{\lambda_{0}}L_{eff}=2\pi \;or\;\lambda_{0}=n_{eff}L_{eff},$$

Here, $n_{eff}=k_{sp}/k_{0}$ is the corresponding effective index of SPP mode confined on the MIM waveguide layer and vertical slit cavity, which strongly depend on the dielectric layer, the air separation, and the metallic nanograting thickness [29,30]. *L*_eff_ denotes the effective cavity length and it is a function of the MIM waveguide and the thickness of the upper and bottom nanogratings. Therefore, it makes sense for the nonlinear dependence of the wavelength of the peak band P_2_ on the upper and bottom nanograting thickness of *t*_u_ and *t*_b_ shown in Figure 2A,B.

Under the incidence of the plane wave at the peak wavelength P_3_ of λ = 1745 nm, the EM field plots are presented in Figure 3C. In this case, the H_y_ field is mostly concentrated inside the layer of the MIM waveguide and forms a nonresonant 1st order waveguide mode along the horizontal direction due to the wavelength much larger than twice of the unit cell period. The intense antinode of the waveguide mode is concentrated on the center of the metallic ridges of the MIM layer, while there is no obvious cavity mode in the vertical slit. The nonresonant peak transmission band is a result of the lager-scale redshift of the 1st order resonant peak band under the modulation of the dip transmission band D_3_ at 1130 nm. Similarly, the formation of the electric dipoles on the input and output of the multispectral plasmonic color filter boosts the light transmission process.

#### 3.2.2. At Dip Transmission Bands

**Figure 4:**
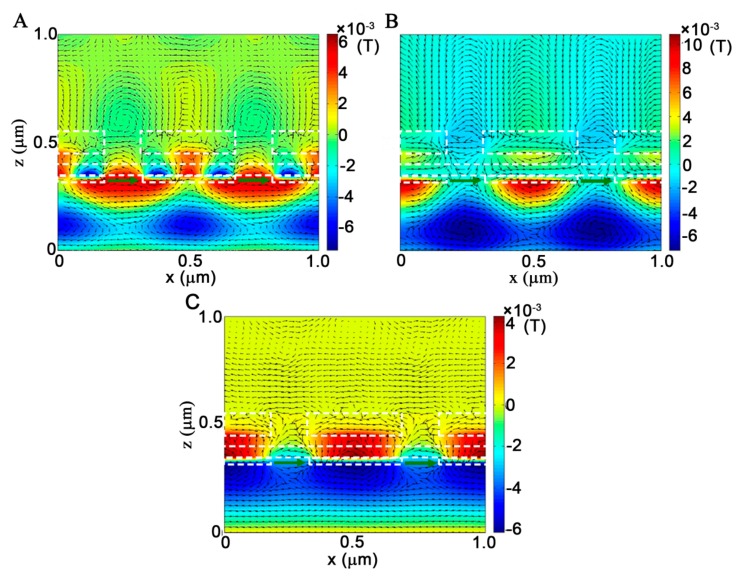
Magnetic field H_y_ (color map) and electric displacement (arrow map) distribution of the plasmonic color filter for the dip transmission band of (**A**) D_1_ at λ =465 nm, (**B**) D_2_ at λ = 530 nm and (**C**) D_3_ at λ = 1130 nm. The parameters of the proposed structure are *t*_u_ = 100 nm, *t*_b_ = 30 nm, *w* = 150 nm, *t*_d_ = *t*_a_ = 50 nm and Λ = 500 nm.

To examine the physical mechanisms of the null-zero dip transmission bands, both the magnetic field (color map) and electric displacement (arrow map) distributions are displayed in Figure 4A–C, which correspond to the dip bands D_1_, D_2_, and D_3_, respectively. For the dip transmission band D_1_ at λ = 465 nm, the H_y_ field is mainly concentrated on the bottom nanograting, where 1st order SPP resonance is formed on the incident interface while the 4th order one is formed on the transmission interface in one periodic cell. Meanwhile, the phases of the H_y_ field on the two bottom nanograting surfaces are completely opposite, indicating the excitation of a complicated hybrid antisymmetric SPP bound mode wave on the bottom nanograting. Such a short-range mode eventually suppresses the light transmission through the upper nanograting, resulting in nearly null dip band D_1_ in the transmission spectrum [29,31]. For the dip transmission band D_2_ at λ = 530 nm, the intensity of the H_y_ field is entirely localized at the metallic ridge on the incident interface of the multispectral plasmonic color filter with a 1st order SPP mode. Although only a small portion of the light can leak through the slit of the bottom nanograting into the MIM waveguide layer, it is completely bounded at the inner metallic wall of the MIM waveguide layer. The blank distribution of the H_y_ field on the transmission interface indicates that there is no light transmission through the multispectral plasmonic color filter. Therefore, the excitation of this SPP bound mode on the incident interface of the multispectral plasmonic color filter eventually reflects the incident light in the backward direction, which reduces the transmission to nearly zero. For the dip transmission band D_3_ at λ = 1130 nm, H_y_ fields can pass through the bottom grating by the excitation of SPP into the MIM waveguide layer to form 1st order SPP mode. More importantly, the SPP mode inside the MIM waveguide is comparable in intensity with that on the transmission interface of the multispectral plasmonic color filter but completely opposite in phase, which demonstrates the formation of a typical antisymmetrical SPP bound mode [28,31]. In addition, the natural property of the short-range propagation makes the SPP bound mode not transmit the light through the upper nanograting, which is responsible for the broad bandwidths of the dip transmission band D_3_. For the dip transmission bands, the plots of the electric displacement distribution manifest that the electric dipoles are only induced on the input slit portion of the multispectral plasmonic color filter, which is helpless for the light transmission. Therefore, the above analyses demonstrate that at each dip transmission band, the SPP bound mode is excited on the bottom nanograting, which causes the reflection and the absorption of the incident light to hinder the light transmission through the multispectral plasmonic color filter, resulting in the near null values of the dip transmission band in the spectrum.

### 3.3. Influence of Structural Parameters on the Transmission Spctrum

#### 3.3.1. Structural Period

**Figure 5:**
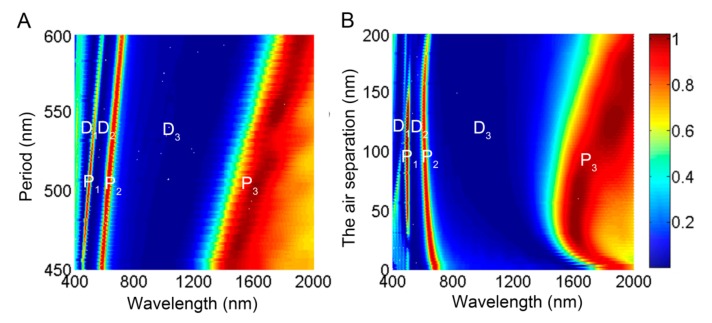
Calculated transmission spectra of the multispectral plasmonic color filter as a function of (**A**) period Λ and (**B**) the air layer thickness *t*_a_.

The influence of the structural parameters, such as the period and the air separation thickness, on the transmission behavior has been also investigated. The spectral transmittance of the multispectral plasmonic color filter as a function of the period is presented in Figure 5A, where the two nanograting thicknesses are adopted as *t*_u_ = 100 nm and *t*_b_ = 30 nm, and other parameters are the same as in Figure 2. Obviously, both the peak and the dip transmission bands show linear red-shift with the increase of Λ. It can be seen that the colors of the peak band vary from violet to red with the change of Λ, which covers the entire visible regime. Because the peak bands in the visible regime respectively originate from the antisymmetric SPP leaky mode undergoing resonance on the transmission interface and the localized SPP mode undergoing waveguide resonance, the linear dependence of the peak bands P_1_ and P_2_ on the period Λ can be inferred from Equations (1) and (2). Furthermore, the dip bands associated with antisymmetric SPP bound mode and localized SPP bound mode are varied as a function of the structural period, and subject to the linear red-shift with the increase of Λ. Therefore, the high and accurate tunability for the positions of the peak and dip transmission bands can be easily accessed by adjusting Λ. Note that the transmission efficiency of the peak band P_1_ reaches the maximum at around Λ = 500 nm and will reduce with increasing or decreasing Λ. The reduction of the transmissivity can be attributed to the nondegenerative energies of the two SPP modes localized on the upper and bottom nanogratings [3], which can be reversed by appropriately modulating the dielectric permittivity and the air layer thickness.

#### 3.3.2. Air Separation

The evolution of the spectral transmittance for the cascaded plasmonic nanograting with the variation of the air separation *t*_a_ is shown in Figure 5B, with the geometrical parameters of *t*_u_ = 100 nm, *t*_b_ = 30 nm, *w* = 150 nm, *t*_d_ = 50 nm, and Λ = 500 nm. For the peak band P_1_ associated with the antisymmetric SPP leaky mode, its wavelength is nearly invariant with increasing *t*_a_ due to the constant period. However, its transmission efficiency drops rapidly to below 25% when *t*_a_ exceeds the range of 50–150 nm. When *t*_a_ is larger than 150 nm, the declined transmission can be attributed to no formation of the hybrid antisymmetric SPP leaky mode, because *t*_a_ exceeds the effective coupling distance (about 150 nm) for the two SPP modes supported by the upper and bottom nanogratings. On the other hand, with *t*_a_ approaching to zero, the dielectric environments surrounding the two cascaded nanogratings are symmetric due to the disappearance of the air separation, the shrinkage of peak band P_1_ results from the nondegeneration of the energies of the two SPP modes on the upper and bottom nanogratings [3]. For peak band P_2_, the wavelength position is slightly blue-shifted while its transmittance gradually reduces with increasing *t*_a_. The blue-shifted phenomenon is ascribed to the reduction of the effective wavelength (or index) of the SPP MIM waveguide mode with the increase of *t*_a_ [30]. The reason for its declined transmittance is also the weakening of coupling between the SPP modes supported by the two cascaded nanogratings. As for the peak band P_3_ in the near infrared regime, its wavelength shifts towards the longer wave with an increase of *t*_a_, which is due to the enlargement of the optical path between the two cascading nanogratings. In contrast to the two peak bands in the visible regime, the transmittances still remain above 90% since the air separation of *t*_a_ = 200 nm is still within the effective coupling distance for the near infrared peak band P_3_, and the corresponding nonresonant SPP mode is still available to travel the incident light through the multispectral plasmonic color filter. Furthermore, this peak transmission band also would be extinct with continuous increase of *t*_a_. The dip transmission bands D_1_ and D_2_, associated with the SPP bound resonance modes on the bottom grating are located in the same wavelength position due to the invariable structural parameters of the bottom nanograting.

For the practical realization of the proposed multispectral plasmonic color filter, its fabrication dimension is supposed to be finite. Referring to the fabrication method of fully suspended slot waveguide and free-standing plasmonic metal-dielectric-metal filter in literature reports [16,32,33], our designed multilayer plasmonic structure can be realized based on the electron beam evaporation, focused ion beam etching, and chemical gas/solution etching technology. During the practical fabrication process, the precise control of the structural parameters, such as the nanograting thickness, the slit width, the air and SiO_2_ layer thickness, the misaligned distance between the upper and bottom nanogratings, etc., are cumbersome to achieve. However, our simulation results show that the designed multispectral plasmonic filter can be tolerant of at least 30 nm errors for the slit width, metallic and dielectric layer thicknesses, and an approximately 40 nm error for the misaligned distance without influence on its spectral performance. If the fabrication error exceeds the critical value, it is no longer suitable for high-efficient multispectral filtering.

## 4. Conclusions

In summary, we have theoretically proposed a novel design of a multispectral plasmonic color filter by cascading two ultrathin nanogratings with two heterogeneous dielectric layers, and theoretically investigated the transmission properties by utilizing the FDTD method. Based on a series of MIM waveguides formed between the two cascaded plasmonic nanogratings, SPP waves confined on the two nanogratings can be overlapped into new hybrid modes, realizing multispectral near-unity peak transmission bands and near-null dip transmission bands. The simulated EM field distributions show that the emergence of the hybrid asymmetric SPP leaky mode and the localized MIM waveguide SPP mode are responsible for two near-unity peak transmission bands, respectively, while the SPP bound modes on the bottom nanogratings can make contributions to the nearly zero dip transmission bands. Remarkably, the high contrast ratio between the peak and the dip transmissions essentially relies on the two heterogeneous dielectric layers sandwiched between the two cascaded nanogratings. This multispectral plasmonic color filter can improve the transmission efficiency up to 90% at the peak bands and perfectly suppresses the light transmission at the dip bands. Moreover, the peak bandwidth can be reduced to as narrow as 20 nm in the visible regime, and the high accuracy multispectral image and sensor in the entire visible spectrum can be accessed by purposefully designing the structural parameters. High-accuracy color filtering performance requires that the spectral resolution imaging analysis capability of the proposed multilayer plasmonic structure is far superior to the human eye and commercial RGB-based color and spectral imaging systems in photo/video cameras. Using a colored pixel to build an ultracompact spectral filtering device, our proposed color filter has potential applications in many fields, such as the color display devices [34], complementary metal-oxide-semiconductor image sensors [18], and light-emitting diodes [35]. The concept of this design can be successfully extended to other wavelengths of the electromagnetic spectrum for multispectral applications.
